# Fecal Microbiota Transplantation for Patients With Irritable Bowel Syndrome: A Meta-Analysis of Randomized Controlled Trials

**DOI:** 10.3389/fnut.2022.890357

**Published:** 2022-05-27

**Authors:** Hui-jun Zhao, Xiao-jing Zhang, Na-na Zhang, Bin Yan, Ke-ke Xu, Li-hua Peng, Fei Pan

**Affiliations:** ^1^Department of Gastroenterology and Hepatology, The First Medical Center, Chinese PLA General Hospital, Beijing, China; ^2^Department of Gastroenterology, National Center for Children's Health, Beijing Children's Hospital, Capital Medical University, Beijing, China; ^3^School of Medicine, Nankai University, Tianjin, China

**Keywords:** irritable bowel syndrome, fecal microbiota transplantation, meta-analysis, randomized controlled trials, global IBS symptoms, subgroup analysis

## Abstract

**Background:**

Gut microbiota has been identified as an imbalance in patients with irritable bowel syndrome (IBS). Fecal microbiota transplantation (FMT) is a novel method to restore microbiota and treat IBS patients.

**Objective:**

To conduct a meta-analysis and estimate the efficacy and safety of FMT for the treatment of IBS patients with subgroup analyses to explore the most effective way of FMT for IBS.

**Methods:**

All eligible studies were searched from PubMed, Embase, Web of Science, and the Cochrane Library through multiple search strategies. Data were extracted from studies comprising the following criteria: double-blind, randomized controlled trials (RCTs) that compared the efficacy of FMT with placebo for adult patients (≥18 years old) with IBS. A meta-analysis was performed to evaluate the summary relative risk (RR) and 95% confidence intervals (CIs).

**Results:**

A total of seven RCTs comprising 489 subjects were eligible for this meta-analysis. Pooled data showed no significant improvement of global IBS symptoms in patients with FMT compared with placebo (RR = 1.34; 95% CI 0.75–2.41, *p* = 0.32). A significant heterogeneity was observed among the studies (*I*^2^ = 83%, *p* < 0.00001). There was no significant evidence of funnel plot asymmetry (Egger's test, *p* = 0.719; Begg's test, *p* = 1.000), indicating no existence of publication bias. Subgroup analyses revealed that FMT operated by invasive routes, including gastroscope, colonoscope, and nasojejunal tube, significantly improved global IBS symptoms (RR = 1.96; 95% CI 1.23–3.11, *p* = 0.004) with heterogeneity (*I*^2^ = 57%, *p* = 0.06) and an NNT of 3 (95% CI 2–14). However, FMT delivered *via* oral capsules showed a negative impact on patients with IBS (RR = 0.56; 95% CI 0.33–0.96, *p* = 0.03) with a low heterogeneity (*I*^2^ = 39%, *p* = 0.2) and an NNH of 3 (95% CI 2–37).

**Conclusion:**

The current evidence from RCTs with all routes of FMT does not show significant global improvement in patients with IBS. However, FMT operated by invasive routes significantly improved global IBS symptoms.

## Introduction

Irritable bowel syndrome (IBS) is one of the most diagnosed GI conditions and a symptom-based functional bowel disorder, characterized by recurrent defecation-related abdominal pain, accompanied by altered bowel habits including stool form and frequency (1). The possible pathophysiological mechanisms include genetics, low-grade bowel inflammation, injured mucosal barrier, increased gut permeability, abnormal bile acids metabolism, aberrant serotonin metabolism, altered motility, visceral hypersensitivity, activated immune response, and central neurologic dysfunction (2, 3).

The current evidence suggests that the gut microbiota could be a significant factor in the pathogenesis of IBS patients who are always identified with dysbiosis. Various studies have proven the difference between the gut microbiota of IBS patients and that of healthy people (4, 5). Increased Firmicutes, decreased Bacteroidetes, and an increased Firmicutes/Bacteroidetes ratio were found in IBS patients (4). The gut microbiota is also related to the severity, symptoms, and subtypes of IBS. The severity of IBS was found negatively correlated with microbial richness, exhaled CH_4_, presence of methanogens, and the prevalence of Prevotella enterotype (6). IBS-D and IBS-M patients had a higher prevalence of Bacteroides enterotype in comparison with the IBS-C patients (6). Infections with some bacterial pathogens like *Campylobacter jejuni, Escherichia coli*, and *Salmonella enterica serovar Typhimurium, Clostridioides difficile*, and *Vibrio cholerae* have been found associated with post-infection IBS (7). Exposure to antibiotics is also considered to increase the risk of developing IBS (8). Additionally, the dysfunction of the bidirectional communication between gut microbiota and the brain, known as the brain–gut axis, is widely regarded as a vital reason for the occurrence of IBS (9).

Due to the impact of gut microbiota on the occurrence and development of IBS, several interventions targeting gut microbiota are commonly used to treat IBS. These include dietary interventions, prebiotics, probiotics, synbiotics, and antibiotics. Except for the traditional interventions above, fecal microbiota transplantation (FMT) provides a creative way to restore the abnormal gut microbiome in patients with IBS directly. FMT has been widely accepted as an effective and safe treatment for recurrent and refractory *Clostridioides difficile* infection (10). However, studies on the role of FMT in IBS are limited and inconsistent. Some current clinical studies confirm the effectiveness of FMT in the treatment of IBS, but some come up with the opposite conclusion. The research on the changes in the gut microbiota of IBS patients after being treated by FMT are also far from consistent. Differences in the dosage, frequency, delivery, and preparation method of donor stool make huge heterogeneity in these studies. Hence, a meta-analysis and systematic review with subgroup analyses were conducted to estimate the efficacy and safety, as well as to explore the most effective way of FMT for the treatment of IBS.

## Methods

### Search Strategy

We first conducted a systematic search of the PubMed, Embase, Web of Science, and the Cochrane Library from inception to January 2022. Then, we manually searched the clinicaltrials.gov and relevant gastrointestinal conferences, including Digestive Disease Week, United European Gastroenterology Week, and American College of Gastroenterology, for relevant trials up to January 2022.

The following medical subject headings (MeSH) or free-text terms were used: “Irritable Bowel Syndrome,” “IBS,” “Syndrome, Irritable Bowel,” “Colon, Irritable,” “Mucous Colitis” (free-text terms) were for IBS; “Fecal Microbiota Transplantation,” “Microbiota Transplantation, Fecal,” “Intestinal Microbiota Transfer,” “Fecal Transplantation,” “Transplantation, Fecal,” “Fecal Transplant,” “Donor Feces Infusion,” “Infusion, Donor Feces” were for FMT. The search results of IBS and FMT were combined using the Boolean term “AND.” HJZ and FP independently reviewed all titles and abstracts for eligibility based on predefined inclusion and exclusion criteria. The searching protocol was restricted to publications with human subjects, but no language limitations.

### Study Selection

The articles included should satisfy the following criteria: double-blind, randomized controlled trials that compared the efficacy of FMT with placebo for adult patients (≥18 years old) with IBS were eligible, including crossover RCTs reporting the data of the first treatment period; patients with IBS should meet the accepted symptom-based criteria (Manning, Kruis, Rome I, Rome II, Rome III, or Rome IV) or a physician's opinion. Studies had to report whether there was a global improvement of IBS symptoms after therapy. Minimum duration of 8-week follow-up was required. When studies did not offer the dichotomous data but were eligible for inclusion, we contacted the first authors or corresponding authors of these studies to obtain additional information.

### Outcome Measures

The primary outcomes were the efficacy of FMT compared with placebo for response to therapy assessed by global improvement in IBS symptoms. Global improvement was defined as a self-report improvement of overall IBS symptoms or the reduction of IBS-related symptom questionnaires, including the IBS Severity Scoring System (IBS-SSS) or gastrointestinal symptom rating scale, IBS version (GSRS-IBS). Secondary outcomes were the change in IBS-specific quality of life (IBS-QOL), adverse events (AEs, total AEs, or individual AEs, including diarrhea, constipation, abdominal pain, bloating, and nausea), and microbiota alterations following FMT.

### Data Extraction

Two authors (HJZ and XJZ) independently extracted all data into a Microsoft Excel spreadsheet. We collected the general information and outcomes from all eligible studies, including country of origin, the number of centers and population, study design, IBS criteria and subtypes, preparation of fecal microbiota and placebo, FMT route and frequency, follow-up, primary outcomes, and AEs as dichotomous data, the change of IBS-SSS and IBS-QOL as continuous data. Dichotomous data were extracted by intention-to-treat analysis and dropouts were regarded as treatment failures. For continuous data, if the studies did not report the mean and standard deviations (SD), they were estimated based on the previous methods (11, 12).

### Assessment of Risk of Bias

Two investigators (HJZ and XJZ) independently performed the quality assessment using the Cochrane Risk of Bias tool (11), and disagreements were discussed with the third investigator (FP). We documented the following information to evaluate the risk of bias of the included RCTs: the generation of randomization schedule, concealment of allocation, blinding of participants, personnel and outcome assessment, incomplete outcome data, selective reporting, and other bias. We further evaluated the quality of evidence using the GRADE (Grading of Recommendations Assessment, Development and Evaluation) method (13).

### Data Analysis

Data analysis was performed using a random-effects model based on the DerSimonian and Laird (14) method. Summary relative risk (RR) and 95% confidence intervals (CIs) were calculated for dichotomous outcomes, including the global improvement, and total and individual AEs. Pooled mean difference (MD) and 95% CIs were reported for continuous outcomes, including IBS-SSS and IBS-QOL. Significant heterogeneity was defined using the Cochrane Q statistic with a *p*-value <0.10 and *I*^2^ statistic with a cutoff of ≥50%. Subgroup analyses were performed to evaluate the effects of different factors on the efficacy of FMT in patients with IBS. Publication bias was assessed by funnel plot, Egger's and Begg's tests (15). The number needed to treat (NNT) or the number needed to harm (NNH) was calculated by the equation: NNT or NNH = 1/[control event rate × (1 – RR)]. RevMan 5.3 (Oxfordshire, United Kingdom) and Stata 14 (StataCorp, College Station, TX) were used for data analysis and to generate plots.

## Results

### General Information, Assessment and Quality

**Figure 1:**
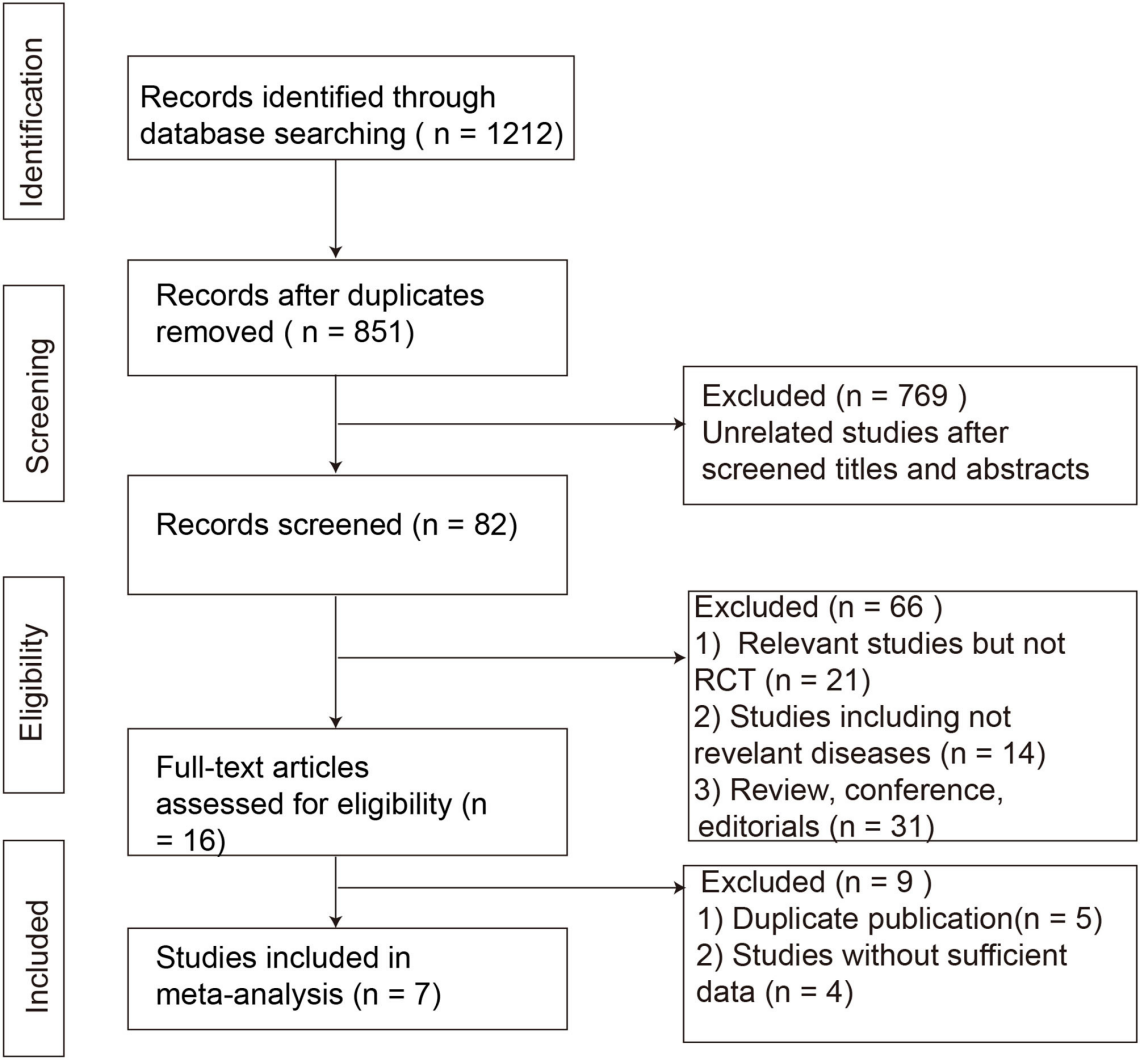
Flowchart of study selection strategy in the systematic review and meta-analysis. RCTs, randomized controlled trials.

A total of 1,212 citations were identified by the combination of key terms (Figure 1). A total of 361 articles were excluded as duplicates, and 769 were excluded as unrelated studies after cross-referencing the titles and abstracts; 66 references were further excluded for various reasons. Finally, 16 full manuscripts were reviewed and only seven RCTs containing 489 subjects were eligible according to the predetermined inclusion criteria (16–22). The general information of the included RCTs is represented in Table 1. A total of six studies (16–19, 21, 22) adopted Rome III as the diagnosis criteria except one study using Rome IV (20). One study included patients with IBS-D only (18), three studies included IBS-D/M (17, 21, 22), and three studies included IBS-D/M/C (16, 19, 20). Among them, two studies performed FMT administration through repeatedly frozen oral capsules with donor stool or placebo-mimics (16, 18), and other five studies through a single invasive method (gastroscopy, colonoscopy, or nasojejunal tube) with the suspension of the donor stool or autologous stool (17, 19–22).

**Table 1:** General information of included seven RCTs.

**References**	**Country**,	**Diagnostic**	**IBS subtypes**	**Sample**	**Allocation**	**Donors**	**Bowel**	**FMT route and location**	**Dosage of**
	**Number of centers**	**criteria**		**size**			**cleansing**	**(upper/lower GI tract)**	**FMT group**
Aroniadis et al. (18)	USA, 3 centers	Rome III	100% IBS-D	48	1:1	Four donors, not mixed	No	Oral capsules, Upper	25 frozen capsules (0.38 g FMT) per day
El-Salhy et al. (20)	Norway,1 center	Rome IV	62 (37.8%) IBS-C; 63 (38.4%) IBS-D; 39 (23.8%) IBS-M	165	1:1:1	One donor, not mixed	No	Gastroscopy, Upper	Frozen 30 g FMT and 60 g FMT
Halkjær et al. (16)	Denmark, 2 centers	Rome III	17 (33.3%) IBS-C; 15 (29.4%) IBS-D; 19 (37.3%) IBS-M	52	1:1	Four donors, mixed	Yes	Oral capsules, Upper	25 frozen capsules (50 g FMT) per day
Holster et al. (19)	Sweden, 1 center	Rome III	4 (25%) IBS-C; 9 (56.3%) IBS-D; 3 (18.8%) IBS-M	17	1:1	Two donors, not mixed	Yes	Colonoscopy, Lower	Frozen 30 g FMT
Holvoet et al. (22)	Belgium, 1 center	Rome III	100% IBS-D or IBS-M	62	2:1	Two donors; not mixed	No	Nasojejunal tube, Upper	Donor fresh feces
Johnsen et al. (17)	Norway, 1 center	Rome III	44 (53%) IBS-D; 39 (47%) IBS-M	90	2:1	Two donors, mixed	Yes	Colonoscopy, Lower	Frozen or fresh 50–80 g FMT
Lahtinen et al. (21)	Finland, 4 centers	Rome III	25 (51%) IBS-D; 7 (14.3%) IBS-M; 14 (28.6%) IBS-unsubtyped; 3 (6.1%) other	55	1:1	One donor, not mixed	Yes	Colonoscopy, Lower	Frozen 30 g FMT
**References**	**Dosage of control group**	**Frequency**	**Follow-up (months)**	**Primary outcome**	**Secondary outcome**				**Microbial analysis**
Aroniadis et al. (18)	25 placebo capsules per day	Multiple: lasting 3 days	3	Difference in the IBS-SSS total score at 3 months	Reduction in the IBS-SSS total score of at least 50 points at 3 months; the assessment of differences in QOL, depression, anxiety, stool consistency and microbiome profiles at 3 months				16S rRNA
El-Salhy et al. (20)	Frozen 30 g autologous feces	Single	3	Reduction in the IBS-SSS total score of ≥50 points at 3 months	Reduction in the dysbiosis index (Di) and a change in the intestinal bacterial profiles at 1 month				16S rRNA
Halkjær et al. (16)	25 placebo capsules per day	Multiple: lasting 12 days	6	Reduction in the IBS-SSS total score of ≥50 points at 3 months	Change in IBS-QOL scores at 3 months and changes in microbiota diversity before and after FMT				16S rRNA
Holster et al. (19)	Frozen 30 g autologous feces	Single	6	Reduction in the GSRS-IBS total score of ≥ 30%	Change of the IBS-SSS, their general health and quality of life (36-item Short Form Survey (SF-36), IBS-QOL, anxiety and depression status				Human Intestinal Tract Chip (fecal and mucosa)
Holvoet et al. (22)	Autologous feces	Single	3	Self-reported improvement of overall IBS symptoms and abdominal bloating at 3 months	Changes in daily assessed IBS symptoms, IBS-QOL, change of IBS-related symptoms scores and fecal microbiota transplantation				16S rRNA
Johnsen et al. (17)	Frozen or fresh 50–80 g autologous feces	Single	12	Reduction in the IBS-SSS total score of ≥75 points at 3 months	Reduction in the IBS-SSS total score of ≥ 75 points at 12 months				NA
Lahtinen et al. (21)	Fresh 30 g autologous feces	Single	3	Reduction in the IBS-SSS total score of ≥50 points at 3 months	Changes in IBS-QOL, gut microbiota, fecal water content, intestinal microbiota composition, and stool dry weight.				16S rRNA

*IBS, irritable bowel syndrome; IBS-C/D/M, IBS with predominant constipation, predominant diarrhea, predominant mixed diarrhea/constipation; GI tract, gastrointestinal tract; FMT, fecal microbiota transplantation; IBS-SSS, IBS Severity Scoring System; GSRS-IBS, gastrointestinal symptom rating scale, IBS version; IBS-QOL, IBS-specific quality of life; NA, not applicable*.

**Figure 2:**
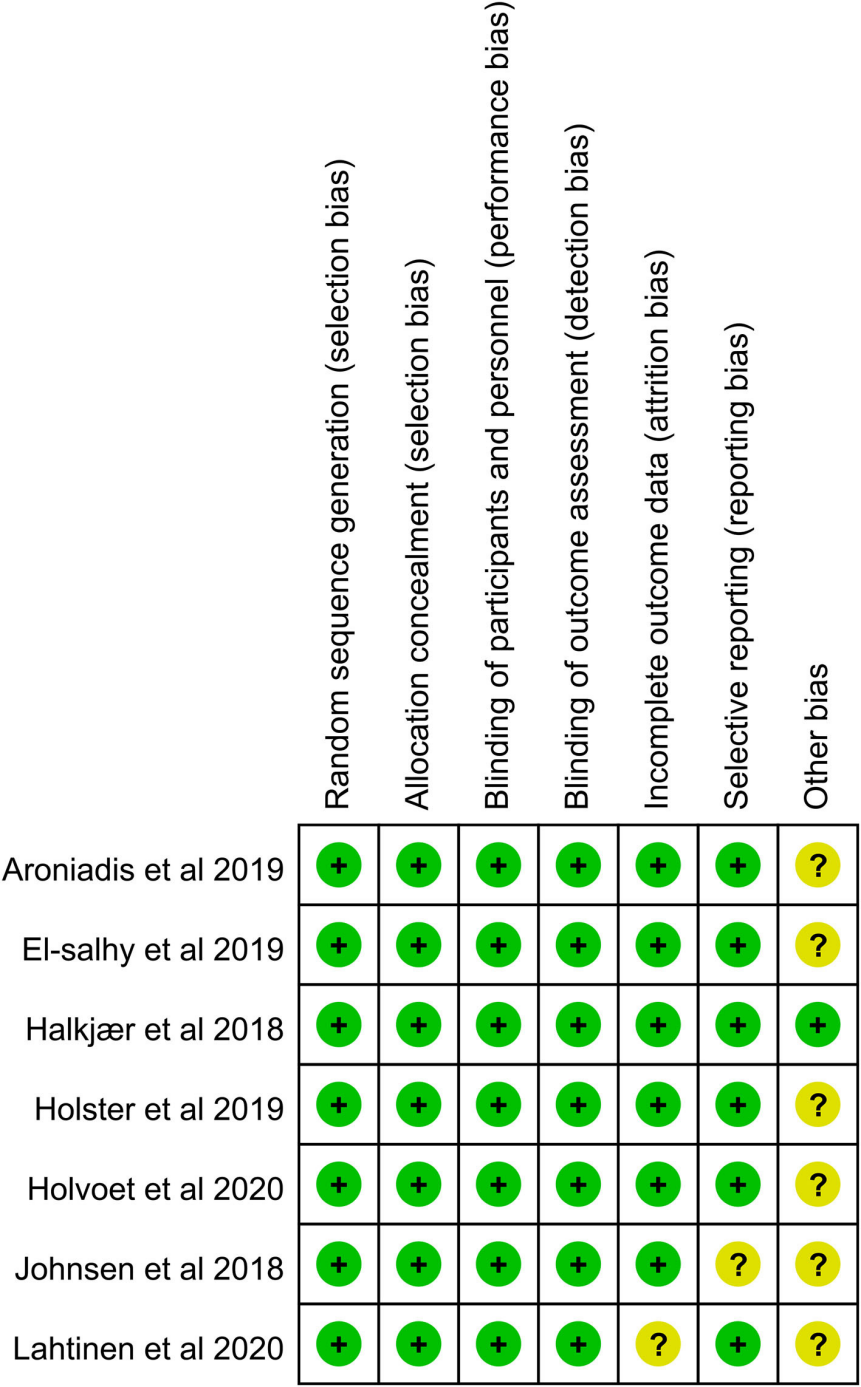
Risk of bias summary.

The risk of bias was summarized using the Cochrane Collaboration tool (Figure 2). In addition, the overall quality of evidence using the GRADE method was “very low” for the inconsistency and imprecision of the primary outcome (Supplementary Table 1).

### Primary Outcome: Global Improvement

**Figure 3:**
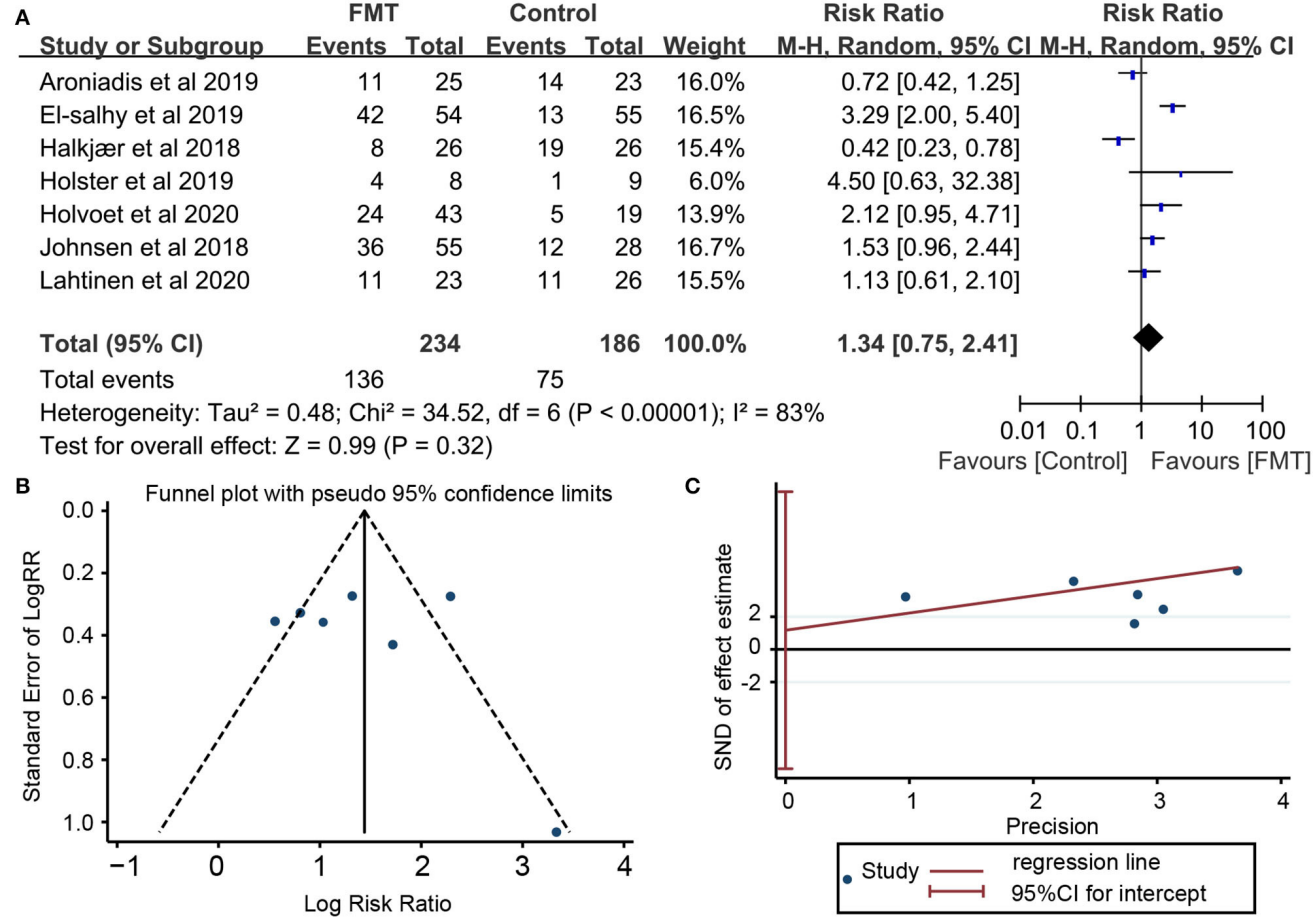
Forest plot and publication bias for the effect of FMT on the primary outcome. **(A)** Forest plot: random-effects meta-analysis of the effect of FMT on the primary outcome of irritable bowel syndrome (IBS) compared with placebo; **(B)** Funnel plot of global improvement of IBS for publication bias; **(C)** Egger's test global improvement of IBS for publication bias.

Seven RCTs involving 420 subjects reported global improvement, which was defined as the reduction of IBS-SSS total score in five trials (16–18, 20, 21), the reduction of GSRS-IBS in one trial (19), or self-reported improvement of overall IBS symptoms and abdominal bloating (22). There were 136 (58.12%) of 234 patients receiving FMT who achieved clinical response, compared with 75 (40.32%) of 186 assigned to placebo. Pooled data showed no significant improvement of global IBS symptoms in patients with FMT compared with placebo [RR = 1.34; 95% CI 0.75–2.41, *p* = 0.32 from random effects] (Figure 3A). A significant heterogeneity was observed among studies (*I*^2^ = 83%, *p* <0.00001). There was no significant evidence of funnel plot asymmetry (Egger's test, *p* = 0.719; Begg's test, *p* = 1.000), indicating no existence of publication bias (Figures 3B,C).

**Figure 4:**
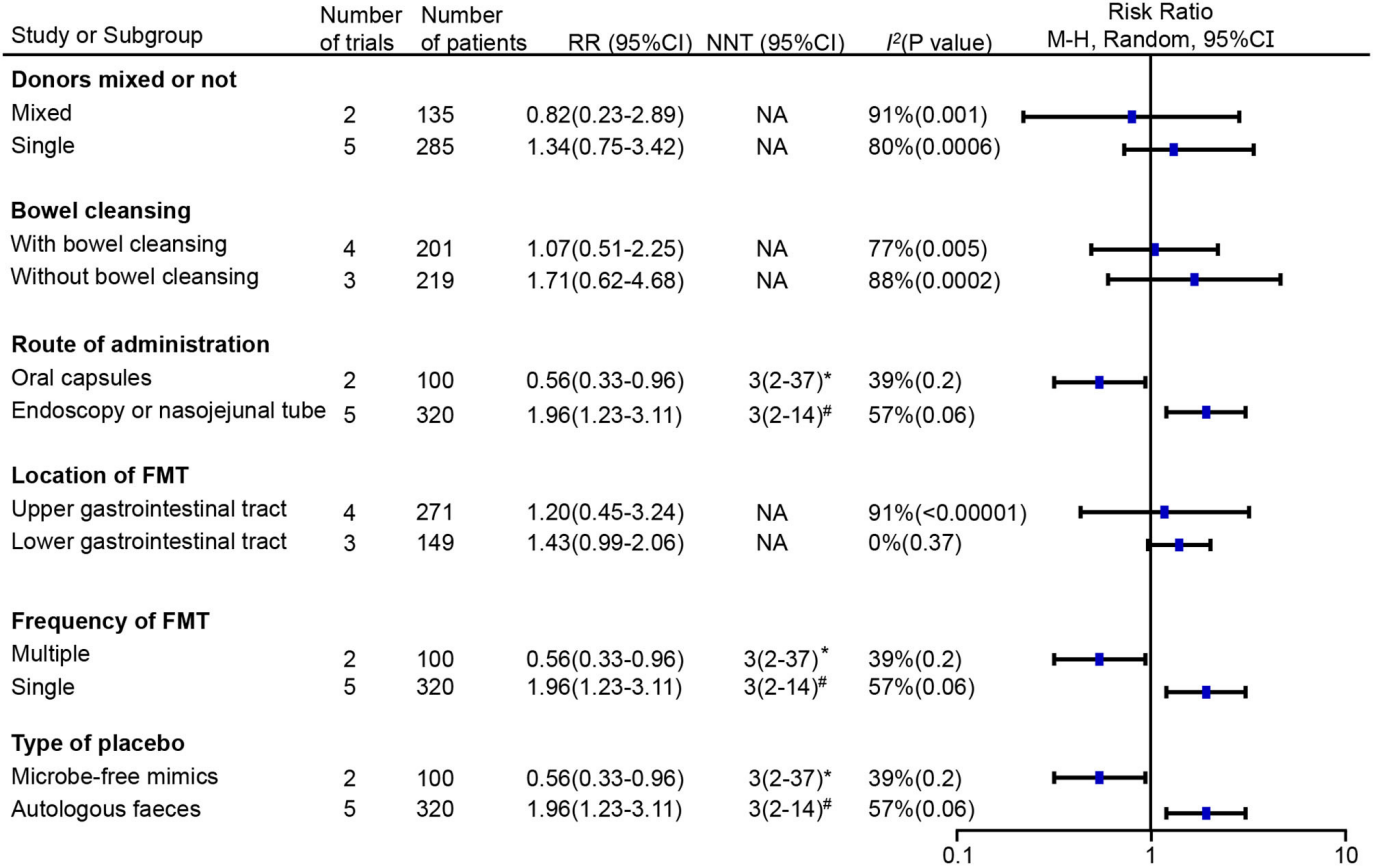
Subgroup analyses of the effect of FMT on the primary outcome of irritable bowel syndrome (IBS) compared with placebo. RR, risk ratio; 95% CI, 95% confidence interval; NNT, number needed to treat; *number needed to harm (NNH); ^#^number needed to treat (NNT); NA, not applicable.

Given the high heterogeneity observed after pooling the results, we further conducted a subgroup analysis based on different designs of administration (Figure 4). FMT operated by invasive routes, including gastroscopy, colonoscopy and nasojejunal tube, significantly improved global IBS symptoms (RR = 1.96; 95% CI 1.23–3.11, *p* = 0.004) with heterogeneity (*I*^2^ = 57%, *p* = 0.06) and an NNT of 3 (95% CI 2–14). However, FMT delivered *via* oral capsules showed a negative impact on patients with IBS (RR = 0.56; 95% CI 0.33–0.96, *p* = 0.03) with a low heterogeneity (*I*^2^ = 39%, *p* = 0.2) and an NNH of 3 (95% CI 2–37). Studies under the invasive routes simultaneously adopted a single infusion of suspension from donor stool in the FMT group or from autologous stool in the placebo group, whereas studies *via* oral capsules adopted multiple doses of capsules from donor stool in the FMT group or from microbe-free mimics in the placebo group. Single FMT was found more beneficial compared to placebo (RR = 1.96; 95% CI 1.23–3.11, *p* = 0.004) with heterogeneity (*I*^2^ = 57%, *p* = 0.06) and an NNH of 3 (95% CI 2–14), whereas multiple FMT was less beneficial (RR = 0.56; 95% CI 0.33–0.96, *p* = 0.03) with a low heterogeneity (*I*^2^ = 39%, *p* = 0.2) and an NNH of 3 (95% CI 2–37). Donor stool was observed more effective than autologous stool (RR = 1.96; 95% CI 1.23–3.11, *p* = 0.004) with heterogeneity (*I*^2^ = 57%, *p* = 0.06) and an NNH of 3(95% CI 2–14), but less effective than microbe-free mimics (RR = 0.56; 95% CI 0.33–0.96, *p* = 0.03) with a low heterogeneity (*I*^2^ = 39%, *p* = 0.2) and an NNH of 3(95% CI 2–37). We further analyzed the influence of donor mixed or not, whether do a bowel cleansing before treatment and the first location of FMT, but no significant effect was observed on the global IBS improvement.

### Secondary Outcome: IBS-SSS and IBS-QOL

**Figure 5:**
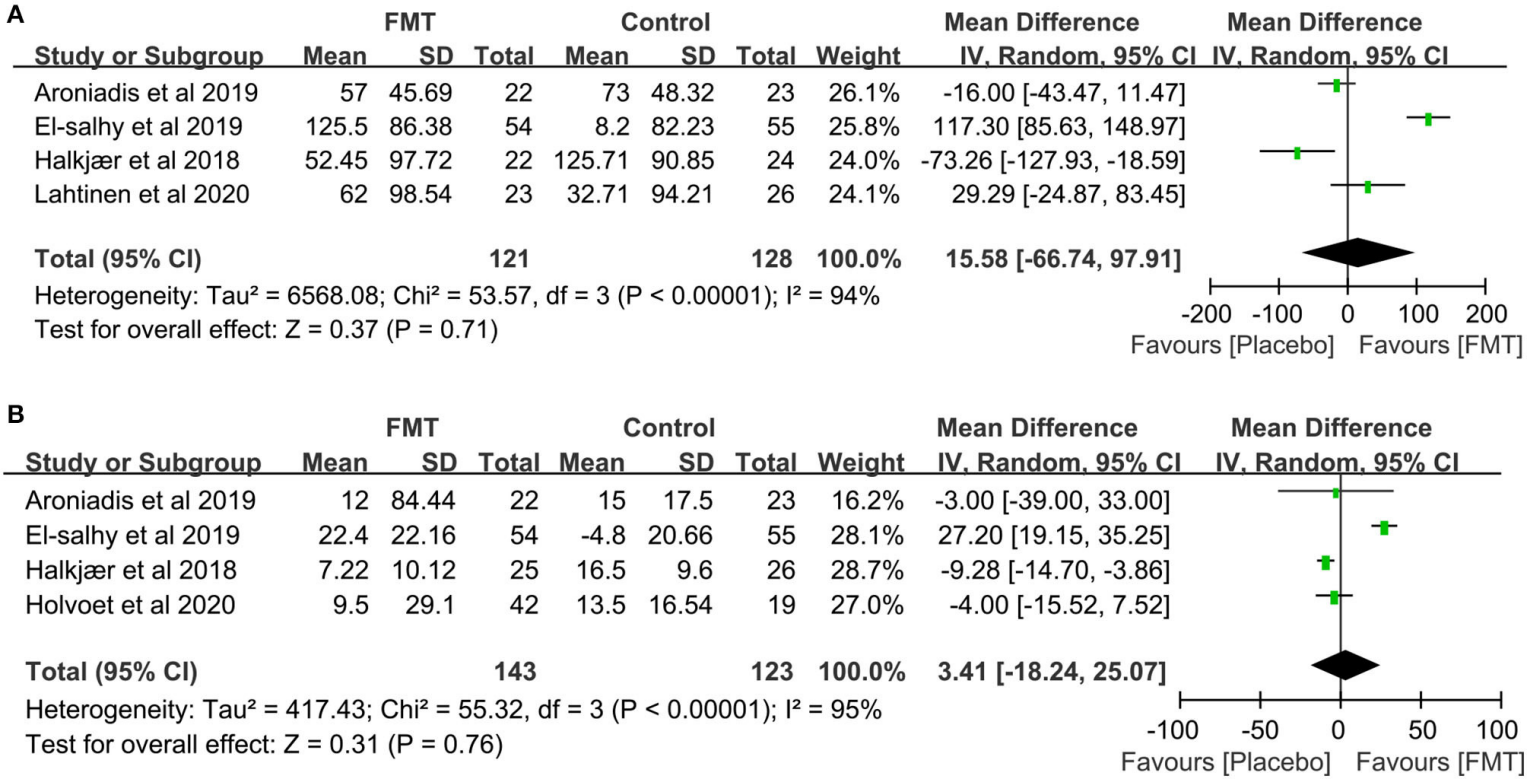
Forest plot of the effect of FMT on IBS-SSS and IBS-QOL. **(A)** Forest plot of the effect of FMT on IBS-SSS compared with placebo; **(B)** Forest plot of the effect of FMT on IBS-QOL compared with placebo.

The change of IBS-SSS was reported in six RCTs (16–21). A total of three trials, El-Salhy et al., Johnsen et al., and Lahtinen et al. (17, 20, 21), showed a significantly improved tendency of IBS-SSS total score in the FMT group compared to the placebo group, especially in El-Salhy et al.'s study (*p* <0.001). A total of two studies (18, 19), Aroniadis et al. and Holster et al., found great improvement in IBS-SSS within groups after treatment, but no difference between groups. Only Halkjær et al. (16) observed a significant relief in the placebo group at 3 months, not in the FMT group (−125.71 ± 90.85 vs. −52.45 ± 97.72, *p* = 0.012). The raw data of IBS-SSS were not available in Johnsen et al. and Holster et al., so we extracted data from the other four RCTs containing 121 participants in the FMT group and 128 in the placebo (16, 18, 20, 21), however, no significant difference was observed between the FMT and placebo groups (mean difference = 15.58, 95% CI −66.74 to 97.91, *p* = 0.71 from random effects, *I*^2^ = 94%) (Figure 5A).

The change of IBS-QOL was compared in six RCTs (16, 18–22). Compared with placebo, one study, El-Salhy et al.'s (20), showed a significant increase in the IBS-QOL total score after FMT. A total of four other studies, Aroniadis et al., Holster et al., Lahtinen et al., and Holvoet et al. (18, 19, 21, 22), observed no dramatic difference between groups. One study, Halkjær et al.'s (16), showed a greater improvement in the placebo group than that in the FMT group. The raw data of four RCTs were available (16, 18, 20, 22) and the pooling analysis, including 143 subjects in the FMT group and 123 in the placebo, showed no significant difference between the FMT and placebo group (mean difference = 3.41, 95% CI −18.24 to 25.07, *p* = 0.76 from random effects, *I*^2^ = 95%; Figure 5B).

### Microbiota Analysis

A total of six of the seven RCTs reported the results of fecal microbiota analyses (16, 18–22). A total of two studies (16, 18) in which FMT was delivered by oral capsules, two studies by colonoscope (19, 21), one study by nasojejunal tube (22), found that the bacterial composition of FMT recipients shifted closer to that of the donors. The study by the nasojejunal tube showed a higher diversity of microbiota in the fecal samples from responders before FMT than that from non-responders (22). However, no significant difference in specific bacteria between responders and non-responders was observed. One study delivered by gastroscope showed that *Eubacterium biforme, Lactobacillus* spp., and *Alistipes* spp. were increased in responders following FMT, and *Bacteroides* spp. was decreased. *Lactobacillus* spp. was negatively correlated with the clinical outcome of IBS-SSS (20).

### Adverse Events

**Figure 6:**
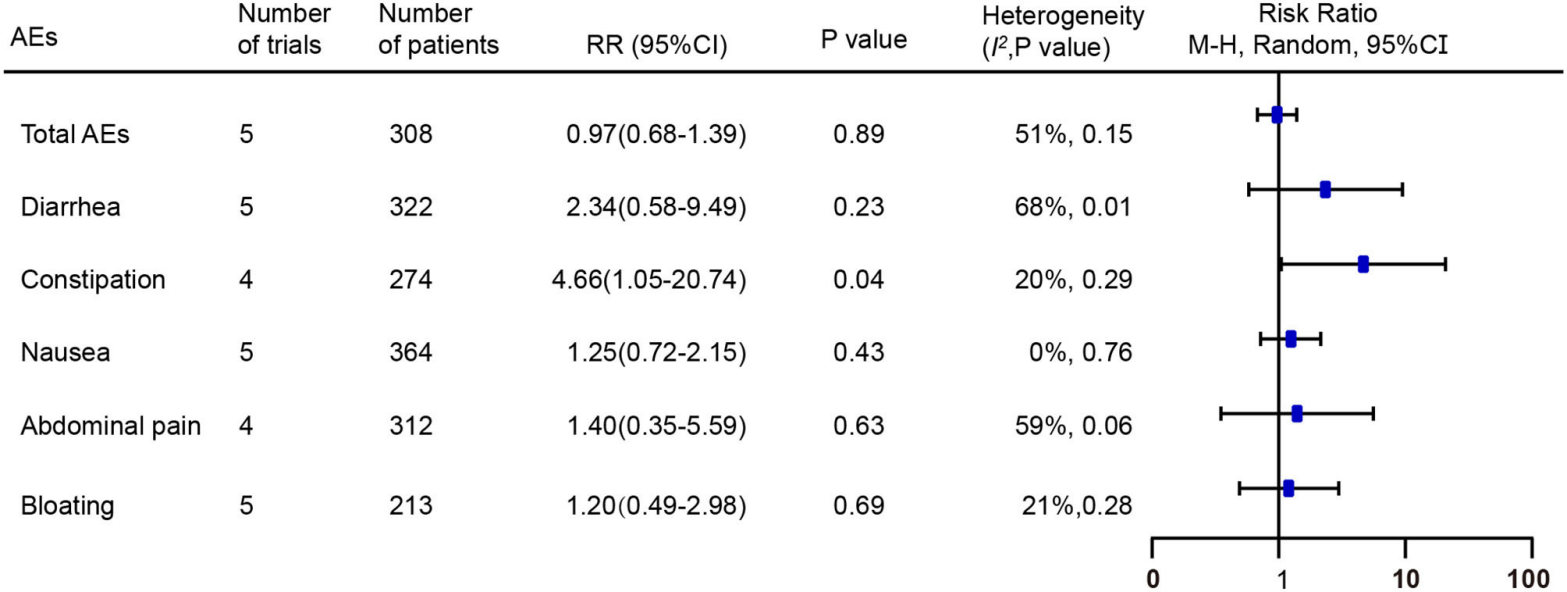
Forest plot of the effect of FMT on total and individual adverse events compared with placebo. RR, risk ratio; 95% CI, 95% confidence interval.

A total of six of the seven RCTs provided the total or individual adverse events (AEs) data (16–21). The total AEs data from five RCTs, including 59 (35.8%) of 165 patients in the FMT group compared with 59 (42.8%) of 138 in the placebo group, were pooled (16–19, 21). No significant difference in the number of total AEs was found between the above two groups [RR = 0.97; 95% CI 0.68–1.39, *p* = 0.89 from random effects, *I*^2^ = 51%]. The most common individual AEs included diarrhea, constipation, nausea, abdominal pain, and bloating. Pooled data of AEs found a higher risk of constipation following FMT compared with placebo [RR = 4.66; 95% CI 1.05–20.74, *p* = 0.04 from random effects, *I*^2^ = 20%] (Figure 6). No significant differences were observed in other common individual AEs. Additionally, Only Johnsen et al. reported that one participant suffered transient vertigo and nausea following FMT, belonging to one serious AE, and needed to be hospitalized for several hours for observation (17).

## Discussion

This systematic review and meta-analysis evaluated the efficacy of FMT in the treatment of IBS and conducted subgroup analyses to explore the influencing factors for the effectiveness of FMT in IBS. According to our findings, the pooled data showed no significant improvement in global symptoms in IBS patients treated with FMT compared with placebo, in line with the conclusions of the previous studies (23, 24). It was shown by pooled data that neither IBS-SSS nor IBS-QOL was reduced by FMT. However, due to the differences in the dosage, frequency, delivery, and preparation method of donor stool, as well as selected donors among the selected research, there has been huge heterogeneity in these studies on the efficacy of FMT in IBS. A total of five of the seven RCTs reported a trend for the bacterial composition of IBS patients to get close to the microbiota of the donor after FMT, including two studies in which FMT was operated *via* oral capsules (16, 18), one study *via* colonoscope (19), one study *via* gastroscope (20), and one study *via* nasojejunal tube (22), revealing the modification of gut microbiota after FMT. As for safety, the pooled data showed a higher risk of constipation after FMT compared with placebo, without showing significant differences in other common individual AEs like diarrhea, nausea, bloating, or abdominal pain. Only one serious AE was reported that one participant suffered transient vertigo and nausea after FMT and needed hospitalized observation (17).

When we further conducted subgroup analysis, a significant improvement of global IBS symptoms was observed in patients treated with FMT *via* invasive routes, including gastroscopy, colonoscopy, and nasojejunal tube, whereas a negative impact was found in IBS patients with FMT *via* oral capsules. In a study by Aroniadis et al., no significant symptom relief was found in the oral FMT group compared with the placebo group (18). Similarly, in the study by Halkjær et al., patients in the placebo group had more obvious symptom improvement than those treated with FMT *via* oral capsules, although increased microbial diversity was observed in the FMT group (16). They further analyzed the effect of oral FMT on abdominal pain, stool frequency, and stool form, but found no clinical beneficial effect (25). In their following study, they found long-term increased anaerobic bacteria in the FMT group, such as *Faecalibacterium, Prevotella*, and *Bacteroides* (26). It is presumed that the changes in the microbiota induced by oral FMT are not significant enough to improve the IBS symptoms. It is likely that invasive FMT can deliver a higher dose of donor stool to patients' bowels at a time than oral capsules, which is supposed to contribute to improving the abundance and diversity of microbiome reaching patients' bowels. A more expansive contact area is available through invasive ways, which may be helpful in the landing of donor stool and the rebuilding of gut microbiota. Considering the things mentioned above, it seems more advisable to choose invasive FMT routes in preference to oral ways.

As for invasive ways for FMT, only in one study by El-Salhy et al., FMT was operated by gastroscopy, using stool from a super donor (20). Definite improvements in abdominal pain, fatigue, and the quality of life were observed in a positive correlation with the dose. Changes in microbiota were observed in this study, like a higher abundance of *Eubacterium biforme, Lactobacillus* spp., and *Alistipes* spp., as well as a lower abundance of *Bacteroides* spp. Relationships between the symptoms and gut microbiota were also detected, such as an inverse correlation between the IBS-SSS score and the concentrations of *Lactobacillus* spp. and *Alistipes* spp., as well as a negative correlation between the Fatigue Assessment Scale (FAS) score and the concentration of *Alistipes* spp. (20). Guo et al.'s study, which was excluded from our meta-analysis for the lack of the standard of global improvement, also reported altered gut microbiota in IBS patients treated with oral FMT (27). Enriched α- and β- bacterial diversity, increased concentrations of the beneficial Bacteroidetes and Firmicutes, as well as decreased toxic releaser Enterobacteriaceae, *Bacteroides*, and *Escherichia coli Shigella* were detected (27), which was partly similar with the result of El-Salhy et al.'s study (20). In El-Salhy et al.'s further study, they detected an increased fecal butyric acid level in the responders to FMT, which could be explained by the above changes in microbiota, indicating that changes in fecal short-chain fatty acids (SCFAs) may be a potential mechanism by which FMT could treat IBS (28).

FMT was conducted by colonoscopy in three studies from Johnsen, Holster, and Lahtinen respectively (17, 19, 21). All of them showed improvements in the IBS symptoms, despite different assessment methods. However, FMT only induced a transient relief for 3 months in accordance with Lahtinen's research, coinciding with the result of Johnsen's study (17, 21). Interestingly, relatively long-term conversions of the composition of gut microbiota were also detected in their studies (21, 29). It may attribute to the complex integrated effects of multiple factors. For example, the low FODMAP diet which IBS patients commonly take can inversely impact the maintaining of functions of planted microbiota, instead of the composition, due to the lack of fermentation substrate. Other factors like drugs and comorbidities may also impact the functions of gut microbiota. But it is a pity that the related information has not been recorded and thus cannot be analyzed.

According to the result of Holster's study, single FMT *via* colonoscopy after bowel cleansing could improve symptoms and quality of life in IBS patients without significant difference compared to autologous FMT (19). Perhaps it could be explained by that bowel cleansing before treatment could improve the restoration of microbiota and the improvement of IBS symptoms after FMT, no matter allogenic or not. There are increasing pieces of evidence for bowel cleansing altering gut microbiota (30, 31). In Freitags' study conducted in mice, pre-interventions with antibiotics before FMT were found useless for the overall plantation of donor microbiota, but helpful for the plantation of *Bifidobacterium*, which was commonly considered as a probiotic (32). However, the specific role of bowel cleansing is still not fully understood. In our subgroup analysis, we also observed that the efficacy of donor stool was superior to that of autologous stool but inferior to that of microbe-free mimics, which is as per that of another meta-analysis from Ianiro G (23). However, when we took all the selected RCTs into account, pooled data showed no significant effect of bowel cleansing before treatment. It was also found that bowel cleansing merely without FMT did not improve the overall restoration of microbiota (32), suggesting that FMT could help rebuild gut microbiota. In Holster's further study, increased expression of the immune-related gene was found induced by allogenic FMT, with a significant difference compared to autologous FMT (33). The utmost response was observed at the time of 2 weeks after FMT, which could partly account for the transient effect of FMT on IBS mentioned above.

Another study conducted by Holvoet et al. in which FMT was operated in the IBS patients with predominant abdominal bloating by nasojejunal tube, reported statistically significant reductions in discomfort, the number of stools, urgency, abdominal pain and flatulence as well as an improvement in quality of life after FMT (22). Additionally, they mentioned high diversity and overall bacterial composition at baseline as an important biomarker to predict successful FMT (22). The effectiveness of FMT was also found positively related to the stability of the microbial composition in the donors and the similarity of the microbiota composition between patients and donors. However, no significant difference in specific bacteria between responders and non-responders was discovered. Repetitive FMT using stool of another successful donor was found effective in a fair number of non-responders to single FMT, suggesting an advantage of multiple FMTs. However, some non-responders still failed to respond to repetitive FMT (22). When we took all the data from selected RCTs into consideration to analyze the influence of FMT frequency on the effectiveness in IBS patients, we even came up with a contrary conclusion to Holvoet's study. It suggests a potential resistance for some IBS patients to FMT. Moreover, it would be a different result after eliminating research in which FMT was conducted *via* oral ways.

We further analyzed the influence on the FMT effectiveness of donor stool mixed or not, but found nothing significant. As for the location of FMT, our subgroup analysis observed no significant effect on the global IBS improvement. However, the study from Ianiro demonstrated a latent benefit for lower-gastrointestinal-tract FMT compared to upper-gastrointestinal-tract FMT (23).

There are some limitations to our study. All the selected RCTs are small-sample studies, thus it is necessary to expand the sample size. Differences in FMT administration routes, doses, frequency, preparation of stools, patient inclusion criteria, and donor selection among RCTs resulted in huge heterogeneity in our study. Therefore, the establishment of a more standard FMT experimental process is still needed. Influencing factors like diet and drugs were not recorded or analyzed in these studies, which may affect the result to a certain extent. Due to the distinct inclusion criteria, it is hard to assess the influence of FMT on various IBS subtypes and symptoms. Thus, larger and more standard RCTs on FMT treating IBS are still required.

In conclusion, pooled data from current RCTs of FMT showed no significant relief for global IBS symptoms, but a lasting alteration of gut microbiota. However, invasive FMT significantly improved global symptoms in IBS patients compared with oral FMT.

## Data Availability Statement

The original contributions presented in the study are included in the article/Supplementary Material, further inquiries can be directed to the corresponding author.

## Ethics Statement

Ethical review and approval was not required for the study on human participants in accordance with the local legislation and institutional requirements. Written informed consent for participation was not required for this study in accordance with the national legislation and the institutional requirements.

## Author Contributions

FP designed research. N-nZ, BY, K-kX, L-hP, and X-jZ performed research. H-jZ and FP analyzed data. H-jZ and X-jZ wrote the paper. All authors contributed to the article and approved the submitted version.

## Funding

This study was supported by the Clinical Research Fostering Fund of Chinese PLA General Hospital (2018FC-WJFWZX-2-15), Science Technological Innovation Nursery Fund of Chinese PLA General Hospital (18KMM02), Young Scholar Medical Research Fund of Chinese PLA General Hospital (QNC19044), Medical Science and Technology Young Scholar Fostering Fund (21QNPY109), and Military Key Discipline Construction Project of Chinese PLA Medical School, 13^th^ Five-year plan (A350109).

## Conflict of Interest

The authors declare that the research was conducted in the absence of any commercial or financial relationships that could be construed as a potential conflict of interest.

## Publisher's Note

All claims expressed in this article are solely those of the authors and do not necessarily represent those of their affiliated organizations, or those of the publisher, the editors and the reviewers. Any product that may be evaluated in this article, or claim that may be made by its manufacturer, is not guaranteed or endorsed by the publisher.
