# Supplementary material for: Fecal Microbiota Transplantation for Patients With Irritable Bowel Syndrome: A Meta-Analysis of Randomized Controlled Trials
Source: Front Nutr. 2022 May 27;9:890357. doi: 10.3389/fnut.2022.890357 (PMC9202577; doi:10.3389/fnut.2022.890357)
Supplement: Supplementary file 1 [file Table_1.docx]

Supplementary Material

# Supplementary Tables

**Supplementary Table 1 GRADE quality of evidence summary of eligible RCTs evaluating the effect of FMT compared with placebo.**

| **Quality assessment** | | | | | | | **Summary of findings** | | | | | **Importance** |
| --- | --- | --- | --- | --- | --- | --- | --- | --- | --- | --- | --- | --- |
|  |  |  |  |  |  |  | **No of patients** | | **Effect** | | **Quality** |  |
| **No of studies** | **Design** | **Limitations** | **Inconsistency** | **Indirectness** | **Imprecision** | **Other considerations** | **Clincal response** | **control** | **Relative (95% CI)** | **Absolute** |  |  |
| **clinical response** | | | | | | | | | | | | |
| 7 | randomised trials | no serious limitations | very serious^1^ | no serious indirectness | serious^2^ | none | 136/234 (58.1%) | 75/186 (40.3%) | RR 1.34 (0.75 to 2.41) | 137 more per 1000 (from 101 fewer to 569 more) | ⊕⊝⊝⊝ very low^1,2^ | CRITICAL |
|  |  |  |  |  |  |  |  | 42.3% |  | 144 more per 1000 (from 106 fewer to 596 more) |  |  |

# ^1^ One study showed a significant influence and other one study showed a negative influence, five studies showed no significant changes.

# ^2^ The total number of subjects is small and we still need more clinical trials.
